# Sensory Lexicon and Major Volatiles of Rakı Using Descriptive Analysis and GC-FID/MS

**DOI:** 10.3390/foods10071494

**Published:** 2021-06-28

**Authors:** Merve Darıcı, Koray Özcan, Duygu Beypınar, Turgut Cabaroglu

**Affiliations:** 1 Department of Food Engineering, University of Çukurova, Sarıçam, 01330 Adana, Turkey; mdarici@cu.edu.tr (M.D.); koray.ozcan@diageo.com (K.Ö.); 2 Mey Alkollü İçkiler San.ve Tic. A.Ş., Büyükdere Cad. Bahar Sok. No:13 River Plaza Kat:25, Şişli, 34394 İstanbul, Turkey; duygu.beypinar@diageo.com

**Keywords:** rakı, volatiles, lexicon, sensory wheel, descriptive analysis

## Abstract

Rakı is a traditional and Protected Designation of Origin (PDO) alcoholic beverage that is distilled from grape distillate with *Pimpinella anisum* L. in copper pot stills in Turkey. This study focused on the development of a sensory lexicon, a sensory wheel, using a consensus approach and the determination of major volatiles by GC-FID/MS for Rakı. A total of 37 Rakı samples representing all producers were used for volatile and sensory evaluation. The experts identified 78 attributes and references for the lexicon. The main attributes were spicy, anise, sweet, resinous, fruity, dry fruit, floral, head&tail aroma and white colour. The Rakı sensory wheel was created to provide a graphical display of its sensory attributes. For validation of the lexicon, 18 samples were evaluated using descriptive analysis. The results were subjected to PCA to examine the relationship of the samples with the defined sensory attributes. The PCA results show that there is a significant relationship between the Rakı categories and sensory terms and flavour intensities. The GC-MS analyses depicted the following major volatile compounds n-propanol, 2-methyl-1-propanol, 2 and 3-methyl-1-butanol, ethyl-acetate, acetal, acetaldehyde, trans-anethol and estragole. The characterization of the product using its most distinctive sensory descriptors are important tool and can be used for the industry, marketing, consumer education and scientists.

## 1. Introduction

Rakı is a traditional distilled beverage produced in Turkey since the time of the Ottoman Empire [1]. Rakı is consumed either neat or mixed with cold water and served with ice in a special long glass. Rakı remains the most produced and consumed distilled alcoholic beverage in Turkey. Its annual production is around 31.2 million litres [2]. Rakı was accorded Protected Designation of Origin (PDO) by the Turkish Patent and Trademark Office in 2009 [3]. According to this PDO document and the Turkish Food Codex Communique on Distilled Alcoholic Beverage [4], Rakı is produced by the distillation of Suma (grape distillate) with aniseed (only *Pimpinella anisum* L.) in a traditional copper pot still that has a maximum volume of 5000 L. Suma is the distillate originating from grapes/raisins that is distilled at up to 94.5% *v/v* alcohol by column still distillation with the purpose of retaining the flavour and smell of grapes. On the basis of its cultural background, traditional method, special composition and flavour, Rakı is a unique and authentic traditional alcoholic beverage produced in Turkey. Turkish regulations define some specific characteristics of Rakı: (a) Rakı should be produced in Turkey; (b) its production should consist of at least 65% fresh grape or raisin-based distillate (Suma) distilled at less than 94.5% volume alcohol with a continuous still to prevent the loss of aromatic compounds, and no more than 35% of the total distillate can be agricultural ethyl alcohol; (c) the amount of anethole—the main essential oil component originating from the aniseed—should be at least 800 mg/L in the product; (d) the volatile substance content should be equal to or exceeding 100 g per hectolitre of 100% volume alcohol; (e) the minimum alcoholic strength by volume of Rakı should be 40% [3,4,5].

The distinctive volatiles that give distilled alcoholic beverages their unique characteristics are affected by many variables such as the raw materials used, flavour additives, and processing steps, which include fermentation, distillation and aging [6]. The volatile components in Rakı determine the sensations of flavour and aroma experienced by the consumer. The general composition and volatile components of Rakı have been reported in previous studies [5,7,8]. These studies showed that Rakı is a rich product in terms of volatile compounds, and its aroma can be assigned to two different origins: Suma (grape distillate) and aniseed (*Pimpinella anisum* L.). It has been determined that anethole and estragole are the major flavour components that come from aniseed [5]. The volatile compounds and sensory properties of Rakı are influenced by many factors, such as the use of fresh or raisin distillate, grape varieties, origin of *Pimpinella anisum* L., amount of aniseed, harvest time of aniseed, storage time of aniseed, distillate origin, distillation technique, number of distillations, maturation, etc. [7]. Normally, Rakı is not a beverage aged in oak barrels. However, short-term barrel aging has been used rarely by some distillers. When the short-term barrel aging process is applied, it contributes to the sensory properties of Rakı.

Sensory evaluation is a key method used to assess the flavour quality of food and beverages. Among the sensory evaluation methods, descriptive analysis has been widely used by researchers to describe the sensory characteristics of food and beverage products [9,10]. This method involves the description of both the qualitative and quantitative sensory factors of products by trained panels [11,12,13,14]. Using quantitative descriptive analysis, a minimum number of terms should provide a maximum amount of information to characterize the product [15,16]. On the other hand, standardized vocabularies known as lexicons can be used to objectively describe the sensory properties of products. Lawless and Civille [17] outlined that a lexicon study is carried out in two stages: preparation and development. The first stage includes the selection of suitable assessors who are highly trained and able to describe the product; the second involves selecting representative samples and developing standardized protocols that the assessors follow. The terms describing the product must then be generated, defined, attached to references, and finalized. Key elements of the lexicon development include a list of the products from which it was developed, all of the attributes and definitions for every attribute with references.

Lexicon flavour wheels or sensory wheels have been developed for many beverages and foods, including wine [18,19], whisky [20], Pisco [21], coffee [22,23], and chocolate [24]. They are widely used in the food industry for sensory quality control, product comparison and monitoring, and to identify distinguishing characteristics of products. The attributes in a sensory wheel serve to standardize training and aid education and discussion. Sensory wheels can serve as a communication tool among all members of the industry, including producers, retailers, exporters, importers, industry professionals and consumers.

To date, no detailed research has been carried out on the sensory profile of Rakı. The main objective of this study was to determine the major volatiles, develop a sensory lexicon for Rakı and group these descriptors and visualize them in a sensory wheel.

## 2. Materials and Methods

### 2.1. Rakı Samples

Commercial Rakı samples (*n* = 37, Table 1) representing the different middle-cut proportions, number of pot-still distillations, and aniseed quantities were obtained from Rakı distillers and retail outlets. The Rakı samples described in Table 1 were gathered from seven distillers in Turkey and included almost all brands belonging to different categories in order to represent the full range of possible sensory attributes. The information used to categorize products was taken from each producer. These categories were related to the suma source, distillate source and proportion, middle-cut proportion, addition of head and tail, number of pot-still distillations, aniseed quantity, price, and quality. When developing the sensory descriptors, it was important to include different ranges of sensory profiles so that many variable sensory descriptors were captured [22,24]. All samples were stored at 20 °C in a dark storage unit.

### 2.2. General Analysis

Alcoholic strength by volume and sugar analysis was carried out according to the OIV reference methods for spirituous beverages of vitivinicultural origin [25]. The determination of alcoholic strength by volume of the samples was carried out by Anton Paar Alcolyzer (DMA 4500M-Alcolyzer ME) with NIR (near infrared) spectroscopy according to OIV reference method (OIV-MA-BS-08:R2009). Determination of sugars in the samples was carried out by HPLC analysis according to OIV reference method (OIV-MA-BS-11: R2009) [25]. The analysis was performed with three replicates for all analysis as shown in Table 1.

### 2.3. Major Volatile Compounds/Direct Injection to GC-FID/MS

Major volatile compounds of Rakı samples were determined by direct injection with a GC-FID/MS according to the European Commission Reference Method [26]. 3-pentanol was used as internal standard. A 0.9 mL aliquot of sample was mixed with 0.1 mL of an internal standard solution (30.86 mg/100 mL in 40% ethanol). Then 1 µL of the above mixture was injected into the GC. As major compounds, acetal, 2-methylbutan-1-ol (active amyl alcohol), 3-methylbutan-1-ol (isoamyl alcohol), methanol, ethyl acetate, butan-1-ol (n-butanol), butan-2-ol (secbutanol), 2-methylpropan-1-ol (isobutyl alcohol), propan-1-ol (n-propanol) and acetaldehyde were determined. GC-FID/MS condition, column, identification and validation parameters of the method were previously reported in detail by Darıcı, Bergama and Cabaroglu [8]. The GC equipment used was an Agilent 6890N, with FID. The CP-WAX 57 CB capillary fused silica column (polyethylene glycol stationary phase, 60 m × 0.25 mm i.d. with 0.4 µm film thickness; Agilent, Netherlands) was used. Injections were made in split mode (split ratio 30:1). Injection temperature was 160 °C and the oven programme was held 4 min at 40 °C, increased from 40 to 94 °C at 1.8 °C/min, then increased from 94 to 180 °C at 30 °C/min, and held 4 min at 180 °C. The FID temperature was 250 °C (H_2_: 30 mL/min and air: 300 mL/min). The carrier gas was helium with a flow rate of 1.3 mL/min. The concentration was determined with respect to the internal standard from the relative response factors (RF) by using FID signal. The identification was performed by comparison with the mass spectrum and injection of reference compounds and the retention times with those of compounds. The results were expressed as g/hL in pure alcohol (PA).

### 2.4. Analysis of Trans-Anethole and Estragole Compounds/Direct Injection to GC-FID/MS

Trans-anethole and estragole were determined by direct injection with a GC-FID/MS according to the European Commission Reference Method (EEC, 2000) [26]. DL-Menthole was used as internal standard. A 2 mL aliquot of the sample was mixed with 2 mL of an internal standard solution (0.19 g/L in 45% ethanol) in a 20 mL flask. Then 1 µL of the above mixture was injected into the GC. GC-FID/MS condition, column and identification and validation parameters of the method have previously been reported in detail by Darıcı, Bergama and Cabaroglu [8]. The GC equipment and the column were the same as described above. Injections were made in split mode (split ratio 40:1). Injection temperature was 230 °C, and the oven was programmed at 180 °C for 15 min. The FID temperature was 230 °C (H_2_: 35 mL/min and air: 400 mL/min). The carrier gas was helium with a flow rate of 2 mL/min. The concentration was determined with respect to the internal standard from the relative response factors (RF) by using the FID signal. The results are expressed as mg/L.

### 2.5. Sample Preparation and Serving

The Rakı samples (Table 1), which were gathered from all distillers in Turkey, were presented in a tulip-shaped wine glass [27] labelled with a random three-digit code. The wine glass used for Rakı sensory evaluations had a scale showing the 25 mL and 50 mL volume lines. Samples (25 mL) were served randomly at 20 °C. Rakı is normally consumed with added tap water at a 1:1 or 1:2 ratio, depending on the consumer’s preference. In each session, 25 mL Rakı samples were served with 25 mL of drinking water at 20 °C. The assessors were served Rakı samples in a tulip-shaped wine glass and tap water separately for each of the Rakı samples. During evaluation, Rakı samples were diluted at a ratio of 1:1. In each session, six samples were served to the assessors. Three-minute breaks were taken between the consumption of each sample. Water and plain crackers were served as palate cleansers.

### 2.6. Panel

A total of 16 expert assessors gathered in the testing room of the International Wine and Spirit Academy (IWSA), which is the Wine and Spirits Education Trust (WSET) course provider. The panel consisted of eight females and eight males aged 27–52 years. All assessors had a minimum of 5 years industry experience and 5 years sensory analysis experience. The group consisted of academic personnel from the Food Engineering Department of Cukurova University and the sensory group of the industry. Members of the sensory group of the industry were selected depending on their participation in the in-house “Sensory Analysis General Training and Authorization Program”, which included a minimum of 2000 h training according to ISO 8586:2012, ISO 5496:2006, ISO 3972:2011 [28,29,30], and depending on their regular participation in sensory evaluations for quality control in industrial production. Academic personnel had a minimum of 240 h experience and training in descriptive analysis, and had regularly participated in sensory analysis research at the university. The study was reviewed and approved by the Çukurova University and informed consent was obtained from each assessor prior to their participation in the study.

### 2.7. Development of Lexicon

The leader of the panel welcomed the assessors and explained that the aim was to determine the sensory descriptors for Rakı in terms of appearance, aroma, taste, mouthfeel and aftertaste. The panel leader made sure that everyone participated and kept the assessors focused on the task.

The expert group came for eighteen sessions over five weeks in the sensory room. In the first five sessions, as a training period, the assessors smelled and tasted the prepared references that were considered to be related to the product flavour on the basis of previous research [5,7,8,20,21]. After the training sessions with the references, the formal panel took place over thirteen sessions, in which the Rakı samples were served. In each formal session, six samples were served to the assessors. Assessors gathered two days in a row each week, with four sessions carried out per week. Two sessions were held each day, one in the morning and one in the afternoon. In the last week, only two sessions were carried out. Every sample from Table 1 was served twice.

Assessors used a consensus process to define the attributes, definitions and references [15,17,22]. According to Lawless and Civille [17], lexicon development is comprised of steps that determine a wide range of products, generating the terms used to characterize the products, and using the references and examples that describe the terms in order to develop the final descriptor list. Based on these steps, the assessors evaluated each sample and noted attributes according to their own vocabulary. Then, they took part in a discussion on each sample.

The group started to evaluate Rakı samples with 25 mL added water (25 mL Rakı + 25 mL water, diluted sample). They first evaluated the appearance and aroma (on the nose), and then tasted, where the aroma (perceived in the mouth retro-nasalretro-nasally), taste, mouthfeel and aftertaste were assessed. They then started to establish, discuss and review the terms and references used. References for each attribute were presented to the assessor and then modified as necessary until the assessors agreed on the references.

### 2.8. Defining the Lexicon and Formation of a Sensory Wheel

The expert group defined 315 attributes following the lexicon development panel. The generated sensory terms were reviewed and rationalized by the expert group. The sensory terms with the same or similar meanings were merged and redundant terms were eliminated. Only consensus attributes related to the samples directly were included in the preliminary descriptors list (*n* = 102). For practical purposes, the final descriptor list for the lexicon were further consolidated based on the frequencies of the terms used by the expert group. Attributes with low frequencies (<5%) were eliminated [24,31]. The final attributes developed for the Rakı lexicon were also used to construct the sensory wheel. All of the final attributes of the Rakı sensory wheel were grouped together to constitute sensible categories based on the indications of the expert group and the expertise of the researchers. The sensory wheel was formed using XLSTAT software (Addinsoft, France) version 2019.4.2.

### 2.9. Descriptive Analysis of Rakı Samples for Validation

A trained sensory panel, using descriptive analysis (DA) as described by Lawless and Heymann [9], evaluated the selected 18 samples in Table 1 depending on their categories (premium, mass and low-end). Assessors were chosen from the panel of the lexicon study. The panel included six females and six males aged 27–52 years. All of the assessors had experience in descriptive analysis and were trained according to ISO-8586 [28].

DA was carried out over eight sessions. The first two sessions were for training, with reference standards prepared for each attribute from the experts’ final attributes list (Table 4). During the training sessions, assessors were able to reduce the number of attributes to 63 due to the simplifying measures taken. Selecting the terms from the lexicon list for particular use is a necessary step in making the specific test practical [22,32]. In the formal sessions (sessions 3–6), the assessors evaluated the intensity of each attribute on a 15 cm unstructured line scale anchored by “low” and “high” intensities. In the four formal sessions, six samples were served, but in the last two sessions, seven samples were served to the assessors. Two sessions were held each day, one in the morning and one in the afternoon. Every sample was evaluated in duplicate. For formal sessions, reference standards were prepared to help the assessors.

The samples were presented in a tulip-shaped wine glass labelled with a random three-digit code. Samples (25 mL) were served randomly at 20 °C. An additional 25 mL of drinking water at 20 °C was served for each of the Rakı samples to dilute the samples at a ratio of 1:1. A two-minute break was taken between each sample. Water and plain crackers were served for palate cleansing.

### 2.10. Statistical Analysis

ANOVA was used to analyse the sensory data from the DA using SenPAQ (Qi Statistics version 6.2) in order to determine differences among samples. The ANOVA model considered products and assessor effects, and the interaction between the product and assessors. The panel performance was also monitored SenPAQ. Principal component analysis (PCA) was conducted with XLSTAT software (Addinsoft version 2019.4.2, France) to show the relationships between the Rakı samples and the attributes.

## 3. Results and Discussion

### 3.1. Major Volatiles of Rakı

Thirty-seven Rakı samples, representing different middle-cut proportions, numbers of pot-still distillations, and aniseed quantities, were obtained from Rakı distillers and retail outlets, and analysed to determine their major volatiles. Table 2 shows the major volatile compounds identified and quantified in Rakı samples by direct injection method with GC-FID/MS. The main suma-based volatile compounds were ethyl-acetate as ester, acetaldehyde and acetal as aldehydes, 1-propanol, 2-methyl-1-propanol, 1-butanol, 2-methyl-1-butanol, 3-methyl-1-butanol as higher alcohols. It is well known that these compounds are formed during the alcoholic fermentation of grape sugars by yeast metabolism [33].

Depending on the amounts of suma-based volatiles, higher alcohols were dominant, followed by ethyl acetate and acetaldehyde. The sum of the amount of higher alcohol compounds ranged from 94.93 to 212.11 g/hL PA in Rakı samples. It has been reported that higher alcohols are quantitatively dominant in the volatile compounds among grape distillate-based beverages such as Rakı and Grappa [5,34]. 2-methyl-1-butanol and 3-methyl-1-butanol (Amyl alcohols), related to sweet coked, kerosene, fusel and burnt odours, are abundant higher alcohols and have been noted to be predictors of sensory character in the distilled beverage [5,34]. A high concentration of 2-methyl-1-butanol and 3-methyl-1-butanol (Amyl alcohols) has a negative effect on the flavour of the distillate [35].

The concentration of ethyl acetate as an ester ranged between 5.68 and 24.69 g/hL PA. It has been reported that the concentrations of ethyl acetate are in the range between 44.4 g/hL and 853.8 g/hL PA for Portuguese Bagaceiras, Italian Grappa, Greek Tsipouro and Spanish Orujo [34,36,37]. Rakı has the lowest ethyl acetate concentration range among grape-based distilled beverages. Ethyl acetate contributes the sensory characteristics of Rakı, with fruity odours depending on its concentration [5,34].

Acetaldehyde is the major aldehyde found in distilled beverages. Its content in the Rakı samples analysed ranged between 1.41 and 9.21 g/hL PA. Cabaroglu and Yılmaztekin [5] reported lower values in 40 Rakı samples, ranging between 0.96 and 3.91 g/hL PA. In grape brandies and Cognac, its concentration ranges from 20 to 25 g/hL PA [38,39]. Acetaldehyde in higher concentration gives an unpleasant odour to the Rakı due to the low threshold values (0.0007–200 mg/L) [5]. Sensory descriptors for acetaldehyde have been reported ranging from nutty and sherry-like to an odour reminiscent of overripe bruised apples [40]. Rakı samples had lower concentrations of aldehydes than those reported in grape-based distilled beverages. This can be explained by the use of column distillation in order to produce suma with less than 94.5% *v/v* alcohol. This type of grape distillate contains lower volatiles than pot still distillation.

The amount of total volatile compounds of the Rakı samples were in the range between 104.71 and 220.93 g/hL PA. According to the Turkish Distilled Alcoholic Beverage regulation [4], the minimum concentration of the major volatile compounds of Rakı must be equal to or exceed 100 g/hL PA. This concentration limit is one of the most important quality control parameters for Rakı in the marketplace.

Methanol is not produced by alcoholic fermentation but formed from the enzymatic hydrolysis of pectin during alcoholic fermentation. Methanol levels were in the range of 19.69–104.63 g/hL PA. The Turkish Food Codex Distilled Alcoholic Beverages Regulation requires a limit lower than 150 g/hL of pure alcohol [4]. All methanol values determined in Rakı samples were below the limits.

An important difference between Rakı and other aniseed-flavoured distilled beverages is the direct use of aniseed (*Pimpinella anisum* L.) for Rakı production. The most abundant aniseed-based volatiles compounds that give an anise-like odour are trans-anethole and estragole, with odour threshold values of 0.073 and 0.016 mg/L, respectively [41]. The trans-anethol concentration of Rakı samples ranged between 1010.0 and 2060.0 mg/L. The trans-anethole concentration should be a minimum of 800 mg/L, according to Turkish Distilled Alcoholic Beverage Regulation [4]. It is an important quality parameter due to its contribution to the characteristic flavour of Rakı. The amount of aniseed used in production plays an important role in the sensory quality of the Rakı.

### 3.2. Lexicon

The 37 Rakı samples represented the full range of possible sensory attributes were tasted to develop a Rakı lexicon. At the beginning of the lexicon study, the assessors defined a total of 315 sensory terms. Then, with further discussion, similar terms were combined by consensus of the assessors during the lexicon development panel, and 102 preliminary descriptive terms were determined (Table 3). From these, 11 terms were related to appearance, 72 were related to aroma and 19 were related to taste and mouthfeel.

Before finalizing the final Rakı lexicon, preliminary terms were eliminated if their frequency of use was below 5% [24,31]. Finally, 78 attributes with their definitions and references were developed for the Rakı lexicon. The final list is shown in Table 4. Overall, there are 12 main attributes, 57 aroma attributes, 13 taste and mouthfeel attributes, and 8 appearance attributes included in the Rakı sensory lexicon.

### 3.3. Sensory Wheel

**Figure 1:**
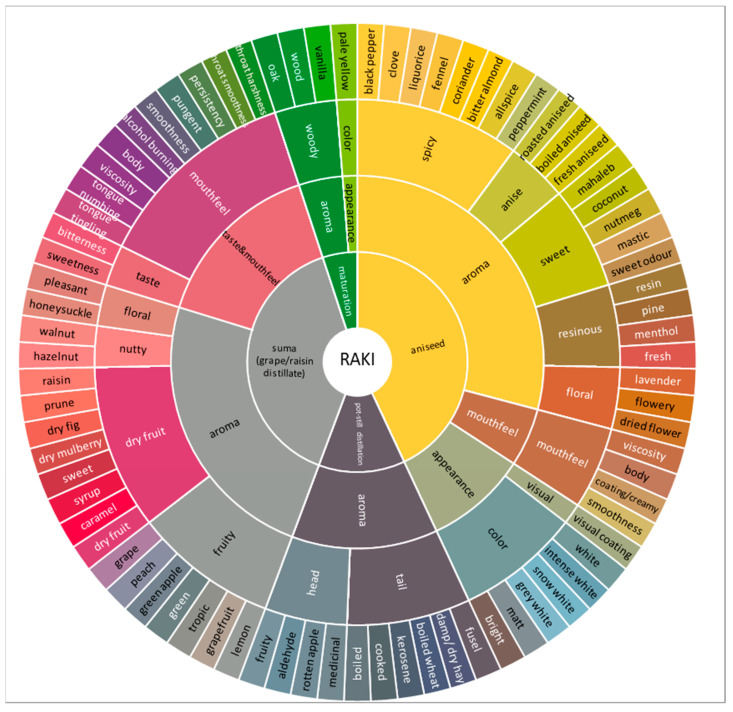
Sensory wheel of Rakı.

A sensory wheel was generated with the help of the final attributes list from the Rakı lexicon. The final attributes of the Rakı lexicon were grouped together to constitute sensible categories based on the findings of the expert group. Eight appearance, 55 aroma and 13 taste and mouthfeel attributes were used for the sensory wheel. A total of 76 specific attributes for Rakı are illustrated in a four-circle wheel (Figure 1).

The inner circle contains the four basic sources of the sensory characteristics of Rakı, namely, suma (grape/raisin distillate), aniseed, pot-still distillation and maturation in barrel. Normally there is no barrel maturation step in Rakı production, but recently, a few producers have begun to use short-term barrel maturation.

In the second circle, the main sensory terms—appearance, aroma and taste and mouthfeel—are given for each source to group the sensory descriptors of Rakı. To describe the appearance of Rakı, eight different attributes were determined: white, intense white, snow white, grey-white, matt, bright and visual coating (Figure 1). Additionally, a pale-yellow attribute was generated for Rakı which indicates barrel maturation. The third circle contains umbrella aroma descriptors determined for the Rakı, which are spicy, anise, sweet, resinous, floral, nutty, fruity, dry fruit, head and tail, and woody. The specific attributes determined in Rakı are given in the outer circle.

### 3.4. Descriptive Analysis to Validate the Rakı Lexicon

A total of 18 selected Rakı samples, of which six belonged to the premium, eight to the mass and four to the low-end categories (from Table 1), were evaluated in the descriptive analysis (DA). For validation of the lexicon, key terms found in most samples were used in the DA. With respect to appearance assessment, assessors evaluated the white colour, visual coating around the glass wall and the pale-yellow colour for samples matured in a barrel. Then, 26 aroma on the nose attributes and 25 aroma in the mouth (retro-nasal) attributes were evaluated. Attributes included alcohol odour on the nose, suma odour, head and tail odour, damp-dry hay, fresh grape, green apple, fruity, raisin, dry fruit, flowery, dry flower, sweet, menthol, spicy, aniseed, roasted aniseed, boiled aniseed, mastic, black pepper, clove, liquorice, fennel, bitter almond, fresh/resin, nutty, woody on nose and in the mouth (retro-nasal) attributes. Sweetness and bitterness were included as taste attributes, and persistency, coating/creamy, body, burning, throat burning, tingling/numbing as mouthfeel attributes. Many of the attributes exhibited significant differences among the Rakı samples (Supplementary Table S1). The attributes roasted aniseed, black pepper, clove, bitter almond, nutty on the nose were not significantly different; therefore, they were not included in the principal component analysis (PCA). The attributes green apple, dry fruit, and alcohol on the nose were included in the PCA due to their importance for Rakı, despite not exhibiting differences between samples.

**Figure 2:**
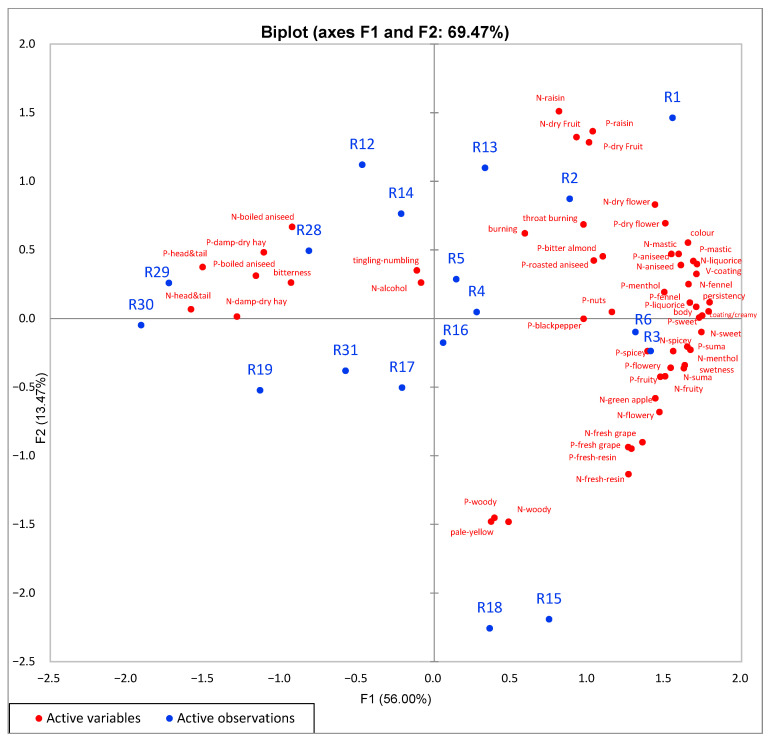
Principal component analysis (PCA) of the trained panel evaluation for appearance (colour and V-coating), aroma on the nose (N), aroma in the mouth (retro-nasal) (P), taste, and mouthfeel attributes of Rakı samples.

Principal component analysis (PCA) was used to evaluate the sensory data gathered from the 18 Rakı samples by trained assessors. PCA results of the 18 Rakı samples are given in Figure 2. In the PCA, variance is explained by the two principal components. The F1 component explained 56.00% of the variance and the F2 component explained 13.47% of the variance.

Appearance was evaluated according to white colour, visual coating and pale-yellow colour attributes in DA. Aniseed has an effect on the appearance of Rakı. Before the preparation of Rakı for consumption, it contains 40–50% alcohol by volume (ABV) and over 0.8 g/L anethole from essential oil that is obtained from aniseed [5]. The aniseed essential oil is soluble at these alcohol levels; consequently, Rakı is colourless in appearance before consumption. However, when it is diluted with water at a ratio of 1:1 or 1:2 for consumption, the colour of Rakı turns white. With the decrease in alcohol level, the essential oil becomes insoluble. With the addition of water, the whitening rate, whitening intensity and hue of Rakı depends on the aniseed quality, quantity and freshness. Furthermore, the essential oil of aniseed makes a coating around the glass wall after the addition of water. The visual coating ratio increases as the aniseed quantity and quality increase. Consequently, the colour and visual coating around the glass wall are sensory attributes related to appearance. It is an important quality characteristic that the colour of Rakı is expected to turn white rapidly and be bright with water addition. Additionally, the visual coating around the glass wall should be apparent and distinctive. According to the appearance characteristics, the PCA shows that Rakı samples separate depending on the colour and visual coating. Pale-yellow attributes were just related with Rakı samples R15 and R18 were matured in barrel.

The Y co-ordinate line separates the R1, R2, R3, R4, R5, and R6 samples classified as premium and containing a high amount of essential oil of aniseed from other samples in Figure 2. The R29 and R30 samples containing a low amount of essential oil of aniseed are present in the upper left side of the coordinate system. R1, R2, R3 and R6, which were premium samples, were highly correlated with colour and visual coating; these samples received high point scores for these attributes. R15 and R18, which were matured in barrels, are grouped together and completely separate from the other samples. These results show that the amount of aniseed and aniseed quality are important factors for the colour and visual coating of Rakı. After dilution with water, aniseed defined the appearance of Rakı.

When the relationships among aroma attributes on the nose/in the mouth and Rakı samples were assessed, it was found that F1 was positively associated with aniseed, sweet, fennel, mastic, spicy, suma, fruity attributes, and was negatively correlated with head and tail, boiled aniseed, damp-dry hay and alcohol attributes. Rakı samples were grouped almost according to their price and distribution categories (low-end, mass and premium) by the PCA. Premium Rakı samples that are produced only from suma and with the addition of high levels of aniseed were associated with a greater number of aroma attributes. One of the premium Rakı samples (R1), produced from raisin distillate and a high level of aniseed, was correlated with raisin, dry fruit, aniseed, mastic and menthol attributes.

Generally, the premium Rakı samples (R1, R2, R3, and R6) were positively correlated with aniseed, sweet, spicy, mastic, menthol, dry fruit, raisin, dry flower, liquorice, fennel, suma, black pepper, flower, fresh grape, and fresh/resin attributes. The low-end samples (R28, R29, and R30) and R19, which was a mass sample, were associated with head and tail, boiled aniseed, and damp-dry hay aroma attributes. Mass category samples R15 and R18 were matured in a barrel for a short time and were associated with the wood attribute along with other aroma attributes. Our results show that the addition of head and tail and agricultural ethyl alcohol reduced the number of aroma attributes and aroma consistency in Rakı samples. It has been reported in previous studies that Rakı is very rich in volatile compounds (propenylphenols, terpenes, higher alcohols, esters, carbonyl compounds), as identified by GC-MS, and that these compounds mostly come from aniseed (*Pimpinella anisum* L.) and suma [5,7,8,42]. Darıcı et al. [8] reported 51 identified aroma compounds and 19 odour active compounds in Rakı, and found that anethole, estragole, linalool, ethyl-2-methyl-butanote, gamma-himachalene, and p-anisaldehyde were the main contributors to the aroma of Rakı. In a study on the determination of a suitable lexicon for the anise-flavoured spirit Ouzo, only eight odour attributes (anise, mastic, sweet, alcoholic, herbs, vanilla, menthol and strong) were reported [43].

In Figure 2, The PCA shows the relationships between mouthfeel and taste attributes of the Rakı samples. Rakı samples R1, R2, R3 and R6, which were in the premium category, were highly positively correlated with persistency, coating/creamy, body and sweetness attributes. The coating/creamy attribute was correlated with the Rakı samples that had a high aniseed oil content. Moreover, two of the premium samples, R4 and R5, were related with the burning attribute. As these samples have the highest alcohol level (%50 *v/v*), the throat burning attribute is a major characteristic that is expected and desirable on some level in Rakı. All of the premium samples and some of the mass samples were positively related with the throat burning attribute. Samples R29 and R30, which were in the low-end category, were negatively correlated with all mouthfeel attributes.

The bitterness attribute was also evaluated in the DA, but it was not an expected characteristic among the Rakı samples. A bitter taste was determined mainly in low-end samples. Due to the excessive recycling with the head and tail cuts in pot-still distillation, bitterness is occasionally perceived from low-end Rakı samples. It has been reported that Pisco—a spirit produced from the distillation of Muscat grape varieties—has parching, burning and mouth-coating attributes as mouthfeel characteristics, and has bitter, sour and sweet attributes as its primary taste characteristics [20]. Tsachaki et al. [43] reported that Ouzo and Tsipouro, which are both anise-flavoured spirits, are associated with descriptors such as sweet, alcoholic, rich, spicy, artificial, aromatic, menthol and caustic; and with aftertaste descriptors including sweet, alcoholic, artificial, spicy and bitter.

## 4. Conclusions

In this study, a lexicon and sensory wheel was developed for Rakı for the first time, the major volatile compounds were determined, and the relationship between the main sensory descriptors and the Rakı samples (according to composition and categories) was investigated. The Rakı lexicon and wheel was developed using 78 and 76 attributes, respectively, covering appearance, aroma, taste and mouthfeel, as determined by the expert assessor group. The sensory lexicon and sensory wheel specify a vocabulary that defines the consensus-constructed attributes of Rakı in order to meet industrial, academic and marketing needs.

Descriptive analysis was used to discriminate between Rakı samples to validate the Rakı lexicon according to their categories (low-end, mass, and premium) in a PCA. The PCA results showed that there was a significant relationship between the Rakı categories and the sensory terms and flavour intensities. It was determined that the ratio of suma, aniseed quantity, distillation program and maturation were the most important factors for sensorial discrimination between Rakı samples. The Rakı samples produced from only suma and containing high levels of aniseed had higher scores than others in the descriptive analysis. On the other hand, the main volatile compounds identified by GC-MS were n-propanol, 2-methyl-1-propanol, 2 and 3-methyl-1-butanol, ethyl acetate, acetal, acetaldehyde, trans-anethol and estragole. Grape distillate-based total volatiles ranged between 104.7 and 220.9 g/hL PA, and anise-based total volatiles ranged from 1029.5 to 2118.6 mg/L in Rakı. This study confirmed that the characteristic sensory properties of Rakı are mainly derived from suma and aniseed. The lexicon can be developed by working with consumers and by adding new attributes over time.

## Figures and Tables

**Table 1:** Main properties of Rakı samples used during the expert panel analysis.

Sample Code ^α^	Price ($) ^β^	Category ^γ^	Suma Source ^δ^	Proportion of Suma	Head & Tail ^ζ^	Alcohol Strength (%ABV)	Sugar Content (g/L)	Application
* R1	27.2	Premium	Raisin	≥65%	nu	47.0	2.0	Triple distillation, 100% raisin Suma
* R2	25.2	Premium	Fresh grape and/or Raisin	100%	√	45.5	4.0	R1, R12, R13 are blended
* R3	30.7	Premium	Fresh grape	100%	nu	47.5	3.0	Triple distillation, %100 fresh grape distillates Suma, produced only in single pot still
* R4	26.9	Premium	Fresh grape	100%	nu	50.0	6.0	%100 Fresh grape Suma, single cycle
* R5	24.5	Premium	Fresh grape	100%	√	50.0	6.0	%100 Fresh grape Suma
* R6	25.9	Premium	Fresh grape	100%	nu	45.0	4.5	Triple distillation, %100 fresh grape Suma, contains 30% roasted aniseed.
R7	174.7	Premium	Fresh grape	100%	nu	50.0	nd	Blend of %100 fresh grape Suma, single cycle Rakı & triple distillation and Rakı %100 fresh grape Suma
R8	28.0	Premium	Fresh grape	100%	nu	45.0	nd	-
R9	25.9	Premium	Fresh grape	≥65%	nu	47.0	8.0	Triple distillation, %100 fresh grape Suma
R10	28.0	Premium	Fresh grape	≥65%	nu	45.0	nd	Triple distillation, %100 fresh grape Suma
R11	28.0	Premium	Fresh grape	≥65%	√	45.0	nd	%100 Fresh grape Suma
* R12	21.3	Mass	Fresh grape and/or Raisin	≥65% ^ε^	√	45.0	4.5	-
* R13	22.6	Mass	Fresh grape and/or Raisin	≥65% ^ε^	√	45.0	4.5	The first harvest of the year aniseed is used in production.
* R14	22.4	Mass	Fresh grape	≥65%	nu	45.0	5.0	%100 Fresh grape Suma
* R15	23.3	Mass	Fresh grape	100%	√	45.0	5.0	%100 Fresh grape Suma, maturing in oak barrels.
* R16	23.3	Mass	Fresh grape	100%	√	45.0	4.0	%100 Fresh grape Suma, contains a small amount Suma from fresh grape of Thracian region.
* R17	19.9	Mass	Fresh grape	≥65%	√	45.0	5.0	%100 Fresh grape Suma
* R18	22.7	Mass	Fresh grape	100%	√	45.0	5.0	%100 Fresh grape Suma, maturing in oak barrels, aniseed grind in stone mill
* R19	18.0	Mass	Fresh grape	≥65%	√	43.0	4.7	%100 Fresh grape Suma
R20	26.0	Mass	Fresh grape	100%	nu	45.0	nd	%100 Fresh grape Suma, maturing in oak barrels.
R21	23.2	Mass	Fresh grape	100%	√	45.0	nd	Maturing in oak barrels.
R22	19.4	Mass	Fresh grape and/or Raisin	≥65%	√	45.0	5.0	-
R23	22.9	Mass	Fresh grape	≥65%	√	45.0	4.5	%100 fresh grape Suma
R24	22.6	Mass	Fresh grape	100%	√	45.0	nd	-
R25	19.4	Mass	Fresh grape	100%	√	45.0	nd	%100 Fresh grape Suma, maturing in oak barrels
R26	19.4	Mass	Fresh grape and/or Raisin	≥65%	√	45.0	nd	Maturing in oak barrels.
R27	19.4	Mass	Fresh grape and/or Raisin	≥65%	√	45.0	nd	-
* R28	19.3	Low-end	Fresh grape and/or Raisin	100%	√	45.0	5.0	Aniseed grind in stone mill
* R29	17.3	Low-end	Fresh grape and/or Raisin	100%	√	43.0	4.0	-
* R30	16.8	Low-end	Fresh grape and/or Raisin	100%	√	42.0	3.0	-
* R31	16.0	Low-end	Fresh grape and/or Raisin	≥65%	√	40.0	3.0	-
R32	16.4	Low-end	Fresh grape and/or Raisin	100%	√	45.0	6.0	-
R33	20.9	Low-end	Fresh grape and/or Raisin	100%	√	43.0	2.0	-
R34	17.1	Low-end	Fresh grape and/or Raisin	100%	√	43.0	nd	-
R35	17.7	Low-end	Fresh grape and/or Raisin	≥65%	√	45.0	nd	-
R36	19.3	Low-end	Fresh grape and/or Raisin	≥65%	√	45.0	1.0	-
R37	15.3	Low-end	Fresh grape and/or Raisin	≥65%	√	43.0	1.0	-

^α^ samples were coded depending on their quality categories; ^β^ prices are given as US dollars using a USD/TRY exchange currency for a 70 cl bottle; ^γ^ category information is taken from each producers; ^δ^ suma, grape or raisin distillate; ^ε^ at least 65% of the total distillate is suma; ^ζ^ samples with √ have partially reused head & tail cuts in pot-still distillations; nu, samples with have no reused head & tail cuts in the pot-still distillation; * Rakı samples with * were used with the trained panel in the descriptive analysis; nd, not detected; all chemical analyses were performed with three replicates and the standard deviation from the mean was under 5%.

**Table 2:** Major volatiles in Rakı samples.

^†^ LRI	Major Volatile	Mean	Min.	Max.	±SE	^‡^ ID
	Suma-based volatiles					
500	Acetaldehyde (g/hL PA)	2.79	1.41	9.21	0.05	MS, LRI, R
898	Ethyl acetate (g/hL PA)	13.97	5.68	24.69	0.19	MS, LRI, R
910	Acetal (g/hL PA)	0.07	nd	1.34	0.01	MS, LRI, R
930	Methanol (g/hL PA)	57.42	19.69	104.63	0.67	MS, LRI, R
1019	2-butanol (g/hL PA)	0.69	nd	4.72	0.04	MS, LRI, R
1047	1-propanol (g/hL PA)	36.05	19.10	60.45	0.35	MS, LRI, R
1074	2-methyl-1-propanol (g/hL PA)	37.61	20.44	88.28	0.49	MS, LRI, R
1130	1-butanol (g/hL PA)	0.48	0.00	1.75	0.02	MS, LRI, R
1204	2-methyl-1-butanol (g/hL PA)	13.79	3.26	24.28	0.21	MS, LRI, R
1204	3-methyl-1-butanol (g/hL PA)	37.10	6.48	73.19	0.61	MS, LRI, R
	Total Volatile Compounds (g/hL PA)	141.61	104.71	220.93	0.92	
	Aniseed-based volatiles					
1823	Trans-Anethole (mg/L)	1370.4	1010.0	2060.0	0.01	MS, LRI, R
1690	Estragole (mg/L)	35.18	19.54	58.56	0.27	MS, LRI, R

Mean, the mean of 37 samples, Min., minimum value; Max., maxiumum value. Each datum is the mean of triplicate determinations; ±SE, standard errors; nd, not detected; PA, pure alcohol; ^†^ LRI, Linear retention indices on DB-WAX column. ^‡^ ID; identification. MS: identification by comparison with the mass spectrum from NIST library. LRI: identification by comparison with data from previous literature. R: identification with the injection of reference compounds.

**Table 3:** Preliminary list of descriptive terms generated during the expert panel for Rakı samples and their frequency of use.

Appearance	F%	Taste/Mouthfeel	F%	Aroma	F%	Aroma	F%
white	31	bitterness	15	spicy	40	flowery	13
intense white	10	sweetness	10	black pepper	7	lavender	5
ghost white	3	astringency	2	clove	7	dried flower	31
grey white	26	tongue tingling	11	liquorice	10	honeysuckle	5
smoke white	2	tongue numbing	11	fennel	10	pleasant	5
snow white	16	alcohol burning	69	coriander	5	fruity	24
pale-yellow	42	viscosity	22	bitter almond	10	lemon	7
yellowish	3	body	12	allspice	10	grapefruit	5
visual coating	31	smoothness	10	peppermint	10	tropic	5
bright	16	pungent	10	roasted aniseed	13	green	7
mat	16	coating	10	boiled aniseed	16	green apple	70
		creamy	10	fresh aniseed	72	peach	5
		coarse	2	sweet odour	61	grape	13
		sharp	2	mahaleb	5	dry fruit	13
		parching	2	coconut	5	caramel	5
		soapy	2	nutmeg	5	syrup	5
		throat smoothness	24	mastic	11	sweet	5
		throat harshness	24	syrup	2	dry mulberry	5
		persistency	65	confectionary	1	dry fig	7
				head and tail	14	prune	7
				fusel	5	raisin	13
				damp hay	13	pine	2
				dry hay	11	medical	2
				boiled wheat	12	kerosene	1
				kerosene	5	earth	1
				cooked	14	moist earth	2
				boiled	10	herbal	3
				medicinal	8	resin	10
				rotten apple	7	pine	7
				aldehyde	5	menthol	15
				rubber	1	fresh	11
				burned tire	2	hazelnut	5
				fusel	3	walnut	5
				boiled wheat	1	woody	6
				chemical	4	oak	5
				Suma	70	vanilla	5

**Table 4:** Attributes and references for the Rakı lexicon.

Main Attribute	Attribute	Definitions	Reference, Preparation
Spicy	Black pepper	spicy and pungent notes associated with black pepper	3 pieces of uncrushed black pepper seeds
	Clove	sweet, spicy, slightly minty, floral notes associated with clove	1 piece of uncrushed cloves
	Liquorice	spicy and sweet notes associated with liquorice	3 g of liquorice root stick
	Fennel	refreshing, light sweet, spicy, anise like notes associated with fennel	3 g of fennel
	Coriander	spicy, soapy and pungent notes associated with coriander seeds	2 pieces of coriander seeds, uncrushed in 25 mL ethanol-water solution (23% *v/v*)
	Bitter Almond	spicy, bitter, sweet, woody and cherry like notes associated with bitter almond	p-anisaldehyde (2 mg/100 mL) in 25 mL ethanol-water solution (23% *v/v*)
	Allspice	spicy, clove and ginger like notes associated with allspice	1 piece of allspice berry, uncrushed
	Peppermint	minty, refreshing, camphor like, notes associated with peppermint	1 drop of peppermint oil in 25 mL ethanol-water solution (23% *v/v*)
Anise	Roasted Aniseed	sweet, herbal, floral, woody notes associated with roasted aniseed	1 dessert spoon of roasted aniseed (*Pimpinella anisum* L.)
	Boiled Aniseed	cooked anise and aniseed aroma and flavour	1 dessert spoon of boiled aniseed (*Pimpinella anisum* L.)
	Fresh Aniseed	sweet, refreshing, herbal, floral notes associated with fresh aniseed flavour	1 dessert spoon of fresh aniseed (*Pimpinella anisum* L.)
Sweet	Mahaleb	bitter-sweet, cherry, nutty like notes related with mahaleb (*Prunus mahaleb*)	1 teaspoon of grinded mahaleb (*Prunus mahaleb* L.)
	Coconut	sweet, slightly vanilla like notes related coconut	1 teaspoon of planed coconut in 25 mL ethanol-water solution (23% *v/v*)
	Nutmeg	sweet, nutty, woody like notes related with nutmeg	1 teaspoon of planed nutmeg in 25 mL ethanol-water solution (23% *v/v*)
	Mastic	sweet, refreshing, elegant, resin, gummy, turpentine-like notes related with mastic	1 teaspoon of crushed mastic (*Pistacia lentiscus* L.) in 25 mL ethanol-water solution (23% *v/v*)
	Sweet Aromatics	confectionary and sweet notes associated with aniseed	1 drop of trans-anethole in 25 mL ethanol-water solution (23% *v/v*)
Resinous	Resin	notes associated with resinous like, pine, fir and fresh notes	1 drop of methyl eugenol (sigma, food grade) in 25 mL ethanol-water solution (23% *v/v*)
	Pine	notes associated with sharp, sweet, refreshing and resinous notes	1 drop of pine essential oil in 25 mL ethanol-water solution (23% *v/v*)
	Menthol	notes associated with minty, cooling, refreshing	1 drop of Menthol essence in 25 mL ethanol-water solution (23% *v/v*)
	Fresh	notes associated with citrus and resinous notes	1 drop of trans-anethole (Merck) in 25 mL ethanol-water solution (23% *v/v*)
Floral	Flowery	sweet, light, slightly fragrant notes associated with fresh flowers	100 uL of natural L-linalool (Sigma-Aldrich) in 25 mL ethanol-water solution (23% *v/v*)
	Lavender	fresh, sweet, floral fragrance associated with lavender	2 pieces of whole fresh lavender flower
	Dried Flower	dry, soft, woody, dusty, floral associated with dry flowers	2 pieces of crushed whole dry lavender flower
	Honeysuckle	sweet, flowery, honey-like and slightly citrus notes associated with honeysuckle flavours	2 pieces of whole honeysuckle flower
	Pleasant	distinctive, flowery and fresh notes related with pleasant	1 drop of rose & honeysuckle essence in 25 mL ethanol-water solution (23% *v/v*)
Tail	Fusel	warm, alcoholic, pungent, ethereal, amyl alcohol odour like associated with tail part of distillation	1 drop of amyl alcohol in 25 mL ethanol-water solution (23% *v/v*)
	Damp/ Dry Hay	dry or damp, slightly sweet, dusty, herbaceous notes associated with hay	Diluted tail part of distillation were used as reference (23% *v/v*)
	Boiled Wheat	boiled grainy, grassy notes associated with boiled wheat	2 g boiled wheat in 25 mL ethanol-water solution (23% *v/v*)
	Kerosene	amyl, solvent, lamp oil and petroleum notes	1 drop of kerosene in 25 mL ethanol-water solution (23% *v/v*)
	Burned	ashy, heavy, heat affected flavours	1 piece of burned rubber
	Boiled	boiled vegetable flavours	3 g cooked cauliflower
Head	Medicinal	solvent, chemical, dusty, drug like flavours	half of a crushed B12 drug tablet
	Rotten Apple	aldehyde like notes associated with rotten apple flavours	3 g rotten apple in 25 mL ethanol-water solution (23% *v/v*)
	Aldehyde	soapy, ripe fruit, waxy and pungent notes associated with aldehyde	1 drop of acetaldehyde in 25 mL ethanol-water solution (23% *v/v*)
	Fruity	mature apple flavours as associated with head fraction	3 g mature apple in 25 mL ethanol-water solution (23% *v/v*)
Suma	Suma Odour	fresh grape and/or raisin distillate flavours (Suma is the distillate producing from grapes/raisin that is distilled at up to 94.5% *v/v* alcohol)	fresh grape suma and raisin suma used as reference
Fruity	Fruity	sweet, fresh, grapes, stone fruit (apple, peaches, pear, cherry) notes blended of a variety of fresh fruity flavours	3 g mixture of shredded apple, pear, apricot, peach in 25 mL ethanol-water solution (23% *v/v*)
	Lemon	citrus, fresh and slightly sweet notes associated with lemon flavours	1 g shredded lemon in 25 mL ethanol-water solution (23% *v/v*)
	Grapefruit	citrus, fresh and sour notes associated with grapefruit flavours	1 g shredded grapefruit in 25 mL ethanol-water solution (23% *v/v*)
	Tropic	pineapple, melon and mango like fruity flavours	3 g mixture of shredded melon, pineapple and mango in 25 mL ethanol-water solution (23% *v/v*)
	Green	sour, fresh and unripe grape flavours	3 g shredded unripe grapes in 25 mL ethanol-water solution (23% *v/v*)
	Green Apple	sweet, sour, bitter, fruity and fresh notes associated with green apple flavours	3 g shredded green apple in 25 mL ethanol-water solution (23% *v/v*)
	Peach	sweet, slightly sour, fruity notes associated with peach flavours	3 g shredded peach in 25 mL ethanol-water solution (23% *v/v*)
	Grape	sweet, fruity, slightly sour notes associated with grape flavours	3 crushed green fresh grapes in 25 mL ethanol-water solution (23% *v/v*)
Dry Fruit	Dry Fruit	sweet and slightly brown notes associated with dry fruit like raisin and prune flavours	3 g mixture of raisin, dried figs and prune in 25 mL ethanol-water solution (23% *v/v*)
	Raisin	concentrated sweet, fruity, jammy notes associated with raisin flavours	2 g shredded raisins in 25 mL ethanol-water solution (23% *v/v*)
	Dry Fig	sweet, rich, lactonic, tobacco like notes associated with dry fig flavour	2 g shredded dry fig in 25 mL ethanol-water solution (23% *v/v*)
	Prune	sweet, overripe, musty, dark fruit notes associated with prune flavour	2 g shredded prune in 25 mL ethanol-water solution (23% *v/v*)
	Dry Mulberry	sweet, overripe, woody and honey-like notes associated with white mulberry flavours	1 g shredded dry mulberry in 25 mL ethanol-water solution (23% *v/v*)
	Caramel	rich, sweet, buttery notes associated with caramel flavours	2 mL caramel solution in 25 mL ethanol-water solution (23% *v/v*)
	Syrup	caramelized sugar, acrid and molasses-like notes associated with syrup flavours	2 mL of sugar beet molasses in 25 mL ethanol-water solution (23% *v/v*)
	Sweet Aromatics	cooked sugar-like flavours associated with sweet substances	1 dessert spoon of boiled raisins in 25 mL ethanol-water solution (23% *v/v*)
Nutty	Hazelnut	nutty, earthy, oily, toasted, cedar notes associated with hazelnut flavours	1 dessert spoon of crushed hazelnut
	Walnut	nutty, earthy, oily, dusty notes associated with walnut flavours	1 dessert spoon of crushed walnut
Woody	Oak	sweet, spicy, woody and dusty notes associated with oak flavour	2 pieces of air-dried French oak chips (Laffort) in 25 mL ethanol-water solution (23% *v/v*)
	Wood	sweet, brown and musty flavours associated with bark of tree	2 piece of tree bark in 25 mL ethanol-water solution (23% *v/v*)
	Vanilla	sweet, spicy, musk and vanilla notes associated with vanilla flavours	1 teaspoon of pure Bourbon vanilla bean powder in 25 mL ethanol-water solution (23% *v/v*)
	Sweetness	The taste factor stimulated mainly by sucrose.	1% sucrose solution as maximum anchor, 0.5% sucrose solution as middle anchor, water without sucrose as lowest anchor
	Bitterness	the taste factor stimulated mainly by quinine and caffeine	0.05% caffeine solution
	Tongue Tingling	pricking, pins and needles sensations on the tongue	Diluted tail part of distillation was used as reference (23% *v/v*)
	Tongue Numbing	loss of sense feeling on the tongue	Diluted fresh grape Suma (20% *v/v*) and diluted Agricultural Ethyl Alcohol, AEA (40% *v/v*) were used as reference
	Viscosity	the thick feel in your tongue by pressing the beverages	1 g carboxymethylcellulose in 500 mL distilled water
	Body	the full, rich, long and heavy feel in your mouth by drinking the beverages	Three anchor Rakı samples were used (R3, R12, R30) depend on their anethol content as reference for body; R3 is highest, R30 is the lowest and R12 is the middle anchor
	Alcohol Burning	burning, warming, irritation sensation on surfaces of mouth when you consume a high-proof of alcohol	Ketel One Vodka and Agricultural Ethyl Alcohol (AEA) were used as reference for alcohol burning; diluted Ketel One Vodka (23% *v/v*) is lowest anchor and diluted AEA (23% *v/v*) is highest anchor
	Pungent	the sharp physically penetration sensation in the nasal cavity	Diluted tail part of distillation were used as reference for pungent attribute (23% *v/v*)
	Coating/Creamy	dense and cream like layer and coat sensation perceived on the tongue	Three anchor samples were used (R3, R12, R30) depending on their anethol content as reference for coating/creamy; R3 is highest, R30 is the lowest and R12 is the middle anchor
	Smoothness	softness, gentleness sensation in the mouth, easy drinking	One of the triple-distilled Rakı samples (R1) was used as reference for smoothness
	Throat Smoothness	softness, gentleness sensation on the throat	One of the triple-distilled Rakı samples (R1) was used for throat smoothness
	Throat Harshness	roughness and puckering sensation on the throat	Agricultural ethyl alcohol (AEA) (23% *v/v*) was used as reference for throat harshness attribute
	Persistency	time that the full sensation sustains in the mouth after swollen	Three anchor Rakı samples were used (R3, R12, R30) depending on their anethol content and suma proportion as reference for persistency; R3 is highest, R30 is the lowest and R12 is the middle anchor
	Visual Coating	appearance, teary, oily and white layer view on the glass wall causing from aniseed oil	Three anchor Rakı samples were used (R3, R12, R30) depending on their anethol content as reference for visual coating; R3 is highest, R12 is the lowest and R30 is the middle anchor
	White	The degree of whiteness, standard white	Rakı sample (R12) has middle anethole content used as a reference for white
	Intense White	The degree of whiteness, deep and yogurt like white	Rakı sample (R3) has highest anethole content used as a reference for bright white
	Snow White	The degree of whiteness, snow like and light white	Rakı sample (R5) has upper middle anethole content used as a reference for snow white
	Grey White	The degree of whiteness, greyish white	Rakı sample (R34) has lowest anethole content used as a reference for grey white
	Matt	dull and flat appearance without brightness	Rakı sample (R34) has lowest anethole content used as the reference for a matt appearance
	Bright	reflecting much light, clear and shining appearance	Rakı sample (R3) has highest anethole content used as the reference for a bright colour
	Pale Yellow	light yellow colour causing ageing in oak barrels	two anchor Rakı samples (R15 and R18), which have a pale yellow colour due to the oak barrel ageing, were used as references; R18 was the lower anchor and R15 was the higher anchor

## Data Availability

The data presented in this study are available on request from the corresponding author.

## Supplementary Materials

The following are available online at https://www.mdpi.com/article/10.3390/foods10071494/s1, Table S1: Result of the Descriptive Analysis.
