# Supplementary material for: Sensory Lexicon and Major Volatiles of Rakı Using Descriptive Analysis and GC-FID/MS
Source: Foods. 2021 Jun 28;10(7):1494. doi: 10.3390/foods10071494 (PMC8306921; doi:10.3390/foods10071494)
Supplement: Supplementary file 1 [file foods-10-01494-s001.zip › foods-1250074-supplementary.pdf]

Table S1. Results of the descriptive analysis.

Attributes	R1	R2	R3	R4	R5	R6	R12	R13	R14	R15	R16	R17	R18	R19	R28	R29	R30	R31	**LSD**	**F**
colour	13.3	11.3	13.6	9.6	9.2	12.6	8.3	9.5	8.3	7.8	8.2	7.5	7.6	7.0	7.6	4.6	5.2	7.8	1.4	***
pale-yellow	0.0	0.0	0.0	0.4	0.0	0.0	0.0	0.0	0.0	8.1	0.0	0.0	8.4	0.0	0.0	0.0	0.0	0.0	0.6	***
V-coating	12.5	9.6	12.9	8.1	7.8	11.6	7.0	8.8	6.8	7.7	6.9	6.2	7.9	5.8	6.1	3.8	4.5	6.4	1.3	***
N-alcohol	4.6	5.4	5.2	6.0	5.4	5.1	5.6	5.6	5.7	5.3	5.3	4.8	5.3	4.8	5.5	5.1	5.2	4.7	1.1	ns
N-suma	5.0	3.9	4.8	4.5	3.9	5.5	3.2	4.0	4.2	4.9	4.4	4.2	4.2	3.2	4.0	2.6	2.6	4.0	1.2	***
N-head&tale	2.3	2.8	2.2	2.6	2.5	1.6	4.4	3.4	3.9	3.4	3.5	3.2	3.2	3.6	3.8	4.5	5.4	3.3	1.2	***
N-damp-dry hay	1.6	2.5	1.9	2.2	2.7	1.4	4.4	3.2	3.8	3.6	3.3	2.7	2.9	2.8	3.1	4.2	3.7	2.8	1.1	***
N-fresh grape	4.0	3.8	5.0	4.7	4.1	4.7	3.4	3.5	4.4	5.9	4.7	4.0	4.9	3.6	3.4	2.8	2.4	3.5	1.2	***
N-green apple	1.8	1.5	2.5	2.1	2.0	2.2	1.5	1.4	1.7	2.2	1.8	1.9	1.8	1.4	1.4	1.0	1.1	1.5	0.9	ns
N-fruity	4.2	4.5	4.5	4.5	4.7	4.8	3.6	3.6	4.2	4.7	4.5	4.3	4.2	3.5	3.2	2.4	3.2	4.4	1.2	**
N-raisin	5.0	4.1	2.9	3.1	3.8	3.5	3.8	3.8	3.6	2.0	2.8	2.7	2.0	2.2	2.6	2.7	2.6	3.0	1.3	**
N-dry Fruit	4.4	3.8	3.0	3.0	3.6	3.4	3.3	3.6	3.5	2.7	2.9	2.8	2.5	2.5	2.7	3.1	2.5	3.2	1.2	ns
N-flowery	4.3	3.6	4.2	4.6	3.5	4.4	3.0	3.4	3.2	4.1	4.1	3.8	4.6	3.5	3.2	3.0	2.2	3.6	1.3	*
N-dry flower	4.2	4.3	4.3	3.6	2.9	3.9	3.6	3.6	3.8	3.2	3.5	3.0	2.9	2.7	3.0	3.0	2.4	2.7	1.2	*
N-sweet	9.9	8.7	10.2	9.1	8.2	9.9	8.1	8.6	7.8	9.1	8.3	7.7	8.8	7.7	7.8	6.2	6.8	7.8	1.1	***
N-menthol	2.7	2.8	3.3	3.0	2.2	3.0	2.2	2.5	2.2	2.9	2.0	2.2	2.5	1.7	1.5	1.6	1.6	1.9	1.1	*
N-spicy	5.6	5.7	5.7	5.2	5.7	6.2	6.0	5.6	5.7	5.8	5.1	5.3	6.1	4.7	4.4	4.4	4.4	5.0	1.1	*
N-aniseed	10.7	9.5	10.8	8.9	8.9	9.7	7.8	9.5	8.2	9.2	8.5	8.4	8.5	8.0	7.5	7.1	6.6	8.2	1.1	***
N-roasted aniseed	0.9	0.9	0.6	0.7	0.7	2.3	0.7	0.5	1.0	0.9	0.7	0.6	0.7	0.4	1.3	0.5	0.4	0.6	1.1	ns
N-boiled aniseed	2.2	2.8	1.9	2.3	2.4	2.1	5.0	3.5	3.4	2.9	3.2	2.5	2.3	2.9	3.7	3.4	3.3	2.2	1.1	***
N-mastic	6.1	4.1	4.8	2.3	1.9	4.7	2.2	2.0	1.7	2.7	2.2	1.8	1.8	1.8	1.3	0.7	1.1	1.3	1.6	***
N-black pepper	2.4	2.7	3.0	2.1	2.6	3.0	2.6	2.6	2.1	2.6	2.2	2.6	2.9	1.8	2.2	1.9	1.7	1.8	1.1	ns
N-clove	1.7	1.8	2.1	1.5	1.9	1.9	1.6	1.6	1.8	1.5	1.5	1.7	1.9	1.3	1.3	1.5	1.1	1.4	0.7	ns
N-liquorice	3.9	2.8	3.6	2.6	3.2	3.0	2.9	3.2	3.2	3.0	2.8	2.8	2.8	2.3	2.6	2.0	1.8	2.4	0.9	**
N-fennel	5.2	4.9	5.0	4.8	4.4	5.3	4.8	4.4	4.9	5.0	4.5	4.5	4.0	3.6	4.0	2.9	3.2	3.9	1.1	***
N-bitter almond	0.8	0.8	1.3	0.6	0.5	0.5	0.7	0.6	0.8	0.6	0.6	0.6	0.5	0.4	0.6	0.3	0.3	0.4	0.5	ns
N-fresh-resin	4.3	4.1	5.2	4.3	3.7	5.0	3.1	3.4	3.5	5.8	4.0	3.4	5.3	3.7	3.1	3.3	3.4	4.0	1.1	***
N-nutty	2.1	2.0	1.7	1.8	1.5	1.9	1.7	1.7	2.0	2.1	1.8	1.8	1.7	1.5	1.5	1.7	1.4	1.4	0.7	ns
N-woody	0.8	0.7	0.9	0.8	0.6	0.7	0.5	0.5	0.8	5.6	0.6	0.8	5.1	0.5	0.5	0.5	0.4	0.6	1.0	***
P-suma	4.5	3.9	4.8	4.3	3.6	4.8	3.1	3.7	4.4	4.2	4.4	4.0	4.2	2.6	3.1	2.6	2.4	3.1	1.0	***
P-head&tail	1.9	2.1	2.1	3.3	2.0	2.0	4.3	3.2	3.9	2.6	3.2	2.7	2.2	3.4	3.3	4.0	4.6	3.0	1.2	***
P-damp-dry hay	1.7	1.7	1.7	2.4	1.9	2.0	4.1	3.2	3.2	2.3	2.5	2.3	2.2	2.5	3.0	3.1	2.9	2.2	1.0	***
P-fresh grape	3.2	4.0	4.7	4.1	3.6	4.0	3.1	3.8	3.9	5.5	4.1	4.0	4.8	3.0	3.2	2.6	2.3	3.2	1.2	***
P-green apple	1.6	1.6	2.2	1.8	1.3	1.5	1.0	1.4	1.6	1.3	1.9	1.7	1.3	0.9	1.3	0.9	1.0	1.2	0.8	ns
P-fruity	4.2	4.0	4.4	4.7	3.9	4.0	3.1	3.8	3.8	4.6	4.5	4.4	3.9	3.0	3.0	2.6	2.8	3.6	1.0	***
P-raisin	5.3	3.9	2.8	3.1	3.3	3.3	3.2	3.3	3.2	2.2	2.2	2.4	1.7	1.9	2.7	2.6	1.7	2.4	1.1	***
P-dry Fruit	4.2	3.8	2.6	2.6	3.3	2.7	3.0	3.0	3.2	2.4	2.5	2.3	2.2	1.9	2.5	2.5	1.9	2.5	1.1	**
P-flowery	3.4	3.1	4.1	3.8	2.4	3.8	2.5	3.5	2.3	3.3	3.5	3.3	3.5	2.3	2.0	2.2	1.6	2.9	1.2	**
P-dry flower	3.8	3.6	3.2	2.3	2.7	3.5	2.6	3.4	3.0	2.9	3.0	2.1	2.6	1.8	2.9	2.1	1.8	2.3	1.1	**
P-sweet	9.2	9.0	9.6	7.8	7.8	8.7	7.0	8.4	7.8	8.6	8.5	7.7	7.9	6.3	6.4	6.0	6.1	7.1	1.1	***
P-menthol	2.6	2.6	2.8	2.6	2.0	3.1	2.2	2.7	2.0	2.6	2.1	1.9	2.2	1.5	1.7	1.5	1.6	2.0	1.1	*
P-spicy	6.0	5.6	5.4	5.3	5.0	6.3	5.8	5.4	4.9	6.1	5.3	5.1	5.9	4.0	4.4	3.9	3.6	4.4	1.2	***
P-aniseed	10.2	9.0	10.1	7.9	8.5	9.2	7.8	8.2	7.9	8.8	8.2	7.6	7.7	6.6	6.4	6.0	6.0	7.2	1.1	***
P-roasted aniseed	1.3	1.3	0.8	0.7	0.8	2.4	0.8	0.7	1.4	0.7	0.9	0.2	0.8	0.5	1.0	0.5	0.6	0.8	0.9	**

P-boiled aniseed	1.8	1.8	1.8	1.9	2.0	1.4	4.0	2.9	3.4	2.7	2.9	2.3	2.0	2.7	2.5	3.2	2.9	2.5	1.0	***
P-mastic	4.8	2.6	3.0	1.5	0.9	3.1	1.0	1.3	1.3	1.6	1.3	1.2	1.7	0.9	1.2	0.6	0.5	1.0	1.2	***
P-black pepper	2.3	3.2	2.1	2.5	2.2	3.4	2.7	2.3	1.9	2.3	1.9	2.2	3.0	1.4	2.3	2.4	1.4	1.8	1.1	**
P-clove	1.6	1.7	1.7	1.3	1.4	1.8	1.6	1.4	1.9	1.8	1.4	1.2	1.5	0.8	1.4	1.4	0.9	1.4	0.7	ns
P-liquorice	3.1	3.4	2.9	2.4	2.5	3.0	2.2	2.9	2.6	2.9	2.6	2.8	2.5	1.9	1.9	1.5	1.2	2.0	0.9	***
P-fennel	4.5	4.3	4.5	4.0	3.9	4.1	3.9	4.3	3.9	4.3	3.8	3.9	4.0	3.1	3.3	2.7	2.4	3.3	1.0	***
P-bitter almond	1.0	0.8	0.7	0.6	0.5	0.2	0.7	0.8	0.7	0.7	0.8	0.4	0.6	0.3	0.5	0.3	0.2	0.3	0.5	**
P-fresh-resin	4.1	3.9	3.5	3.4	3.6	4.1	3.0	2.7	3.4	5.1	3.0	3.2	4.8	2.7	2.8	2.7	2.7	3.5	1.0	***
P-nutty	1.9	2.5	1.6	1.4	1.7	1.6	1.7	1.6	1.7	2.2	1.9	1.5	1.8	1.2	1.5	1.1	1.2	1.2	0.7	**
P-woody	0.8	0.9	0.8	0.6	0.6	0.7	0.9	0.5	0.7	5.1	0.8	0.7	5.0	0.5	0.8	0.9	0.5	0.7	1.1	***
tingling-numbing	4.7	4.6	4.5	6.8	4.4	4.5	4.7	5.1	5.1	4.4	4.9	4.4	5.0	3.7	5.9	5.0	5.1	4.1	1.5	*
body	10.2	8.8	10.1	7.8	7.0	9.9	6.8	7.5	7.4	8.4	7.1	6.0	8.1	5.5	6.1	4.3	4.4	6.2	1.3	***
burning	6.3	5.5	6.0	7.7	5.7	5.1	5.3	6.2	6.5	5.2	5.6	5.6	5.5	4.6	6.4	5.3	5.1	5.1	1.5	ns
coating/creamy	8.5	6.7	8.6	6.3	5.7	7.7	5.8	5.7	5.5	6.9	5.6	4.9	6.5	4.5	4.9	3.9	4.2	4.9	1.2	***
throat burning	6.2	6.3	6.0	7.3	6.6	5.6	6.2	6.4	6.7	6.0	5.9	6.0	5.2	5.2	6.1	5.1	4.6	5.3	1.5	ns
persistency	11.2	9.6	10.8	9.7	8.5	10.3	8.0	9.1	8.8	9.3	8.1	7.6	9.3	5.9	7.2	5.5	5.0	7.1	1.2	***
bitterness	1.8	1.9	1.9	1.2	2.0	2.4	2.2	2.4	3.1	2.0	2.2	2.1	2.2	1.6	2.5	3.2	3.2	2.0	1.1	*
sweetness	7.8	7.6	8.5	7.2	7.2	7.9	7.4	6.5	7.1	7.6	7.3	6.7	7.6	6.3	6.4	6.2	6.2	6.9	1.1	**

F: Significance at which means differ as shown as analysis of variance, *p < 0.05 level, **p < 0.01 level, ***p < 0.001 level; ns: not significant; LSD: Least Significant Differences is calculated at 95% confidence level with the interaction as the error terms. If the difference between the means in the same row is greater than the LSD value, the difference is statistically significant. N; Aroma on the nose and P; Aroma in the mouth (retronasal)
